# Material Considerations for Fused-Filament Fabrication of Solid Dosage Forms

**DOI:** 10.3390/pharmaceutics10020044

**Published:** 2018-04-02

**Authors:** Evert Fuenmayor, Martin Forde, Andrew V. Healy, Declan M. Devine, John G. Lyons, Christopher McConville, Ian Major

**Affiliations:** 1 Materials Research Institute, Athlone Institute of Technology, Dublin Road, N37 HD68 Athlone, Ireland; e.fuenmayor@research.ait.ie (E.F.); m.forde@research.ait.ie (M.F.); andrewhealy@research.ait.ie (A.V.H.); ddevine@ait.ie (D.M.D.); slyons@ait.ie (J.G.L.); 2 School of Pharmacy, Institute of Clinical Sciences, College of Medical and Dental Sciences, University of Birmingham, Birmingham B15 2TT, UK; c.mcconville.2@bham.ac.uk

**Keywords:** 3D printing, fused-filament fabrication, additive manufacturing, hot-melt extrusion, formulation, melt-blending, solid dosage forms, tablets

## Abstract

Material choice is a fundamental consideration when it comes to designing a solid dosage form. The matrix material will ultimately determine the rate of drug release since the physical properties (solubility, viscosity, and more) of the material control both fluid ingress and disintegration of the dosage form. The bulk properties (powder flow, concentration, and more) of the material should also be considered since these properties will influence the ability of the material to be successfully manufactured. Furthermore, there is a limited number of approved materials for the production of solid dosage forms. The present study details the complications that can arise when adopting pharmaceutical grade polymers for fused-filament fabrication in the production of oral tablets. The paper also presents ways to overcome each issue. Fused-filament fabrication is a hot-melt extrusion-based 3D printing process. The paper describes the problems encountered in fused-filament fabrication with Kollidon^®^ VA64, which is a material that has previously been utilized in direct compression and hot-melt extrusion processes. Formulation and melt-blending strategies were employed to increase the printability of the material. The paper defines for the first time the essential parameter profile required for successful 3D printing and lists several pre-screening tools that should be employed to guide future material formulation for the fused-filament fabrication of solid dosage forms.

## 1. Introduction

Three-dimensional printing (3DP) is finding increasing utility in the manufacture of pharmaceutical dosage forms [1]. The production process is available in some different formats [2], but it can be best defined as a process that creates a physical object from a digital model through the layer-by-layer deposition of material. Potential pharmaceutical applications for 3DP are diverse and span conventional dosage forms such as tablets and implants as well as less typical pulsatory devices [3]. Spritam^®^ is the first FDA approved 3D printed dosage form and is an orodispersible tablet containing different doses of the anti-seizure drug levetiracetam [4]. The manufacturers harness 3DP to enable high dosing (1000 mg) while maintaining a highly porous structure that aids rapid disintegration necessary for orodispersibles. Innovation lies at the heart of the drive towards 3D printed dosage forms meant for both the creation of more complex devices and to meet the requirements of on-demand manufacturing and precision medicine [1].

Fused-filament fabrication (FFF) is a method of 3D printing that incorporates the hot-melt extrusion (HME) process for manufacturing a thermoplastic filament that can be fed directly to an extrusion head for melt-deposition in a pre-programmed x-y-z axis. The process is also commonly known by the trademarked name Fused Deposition Modelling™. HME is a well-established process for the production of pharmaceutical dosage forms, which ensures solvent-free production, low-cost scale-up, and enhanced solubility for poorly water-soluble drugs [5]. FFF is a mainstay among 3DP amateur enthusiasts since the printers are relatively inexpensive and the filaments come in an abundance of colors. The technology is gaining interest in pharmaceutical research groups worldwide that were previously described in the production of a variety of different experimental dosage forms [6]. Producing drug-loaded filaments to strict tolerance of diameter and shape can be slow and cumbersome without the prerequisite HME processing equipment. For this reason, some groups opt to impregnate the commercial filament with the drug [7,8,9] while others can produce drug-loaded filaments for FFF via the HME process to print experimental tablets [7,10,11,12,13,14,15,16,17,18,19], caplets [20,21], implants [22,23,24], discs [25,26,27], and topical devices [28].

Material choice is a fundamental consideration when designing a pharmaceutical dosage form. Physical properties of the material will influence the rate of release and the type of release with the matrix polymers ranging from fully biodegradable [29] to non-degradable [30]. Material bulk properties will also deter the route of manufacturing since certain properties can exclude particular processes. A further restriction is a limited range of polymer materials with regulatory approval as excipients for the production of solid dosage forms [31]. For FFF in general, the range of materials available is very narrow compared with other conventional HME based processes. Thirty materials available commercially were compared to over three thousand for the other HME-based processes [32]. There is currently no formalized method for pre-screening materials for the FFF process [33] and there is no definitive property profile of FFF feedstock in the literature for benchmarking materials for printability. A number of pharmaceutical materials have been investigated for FFF including polyvinyl alcohol [7,8,9,11,12,17,20,21,27], cellulose-based polymers [13,19,24,27], polylactic acid [24,25,27,28], polycaprolactone [22,24,28], ethylene vinyl acetate [23], polyvinylpyrrolidone [15,16], Soluplus^®^ [26,27], Kollicoat^®^ IR [27], and Eudragit^®^ grades [14,18,24,26,27].

The initial concept of this work was to manufacture oral tablets by direct compression, injection molding, and FFF for comparison. Kollidon^®^ VA64 (PVP-VA) was chosen as the polymer matrix material since it is recommended for both direct compaction and HME by the manufacturers BASF [34]. We have previously reported on the use of PVP-VA in the production of both direct-compaction [35] and injection molded [36] multi-layered vaginal tablets in which the polymer constituted the immediate release layers. However, our initial trials highlighted some issues including filament brittleness and stiffness, which prevented the simple adoption of this polymer for the FFF manufacture of similar tablets. Similar to other pharmaceutical grade polymers [26], PVP-VA has inherent limitations that prevent FFF based 3DP. These will be discussed in detail. In a wider context, the same issues will be relevant for similar pharmaceutical grade polymers without the necessary set of physical properties required for successful FFF-based 3DP. This present work aims for the first time to detail the necessary material property profile for a pharmaceutical grade polymer to successfully undergo the FFF process for complete-batch runs, which is defined as a batch that utilizes the full working surface of the print-bed without operator interference. There is a focus on the inherent compromises that must be made between printability and final dosage form properties. We outline potential difficulties faced at every stage of the FFF process and list suitable strategies to deal with each issue. We describe a melt-blending approach that can be adapted by other researchers to accommodate their specific needs from a matrix material and simultaneously overcome material shortcomings that prevent FFF 3DP.

## 2. Materials and Methods

### 2.1. Materials

Kollidon^®^ VA64 and Kolliphor^®^ P188 were purchased from BASF Ireland (Cork, Ireland). Poly (ethylene oxide) (PEO) (average *M*_WT_ = 300,000) in a white powder form was obtained from Sigma-Aldrich (Arklow, Ireland). Polycaprolactone (PCL) in powder form (CAPA 6506, average *M*_WT_ = 50,000) was obtained from Perstorp (Cheshire, UK). USP grade caffeine was purchased from VWR International (Dublin, Ireland). Caffeine was chosen as a Biopharmaceutical Classification System (BCS) Class I model drug since it was available in sufficient quantities to complete the overall study (of greater scope than this paper), has a melting temperature greater than the processing temperatures, and was safe for use in the environments the production equipment were located. Table 1 displays all placebo formulations manufactured in this body of work for their mechanical characterization and for pre-screening trials.

Five formulations were produced with the model drug caffeine and can be found in Table 2. These mixtures were produced to perform drug dissolution studies to evaluate the effects of the formulations and PVP-VA loading on drug release kinetics as well as to compare tablets that were manufactured via direct compression and 3DP.

### 2.2. Preparation of Filaments by HME

Thirteen material formulations are outlined in Table 1. Before HME processing, all excipients were passed through a 450 µm sieve to obtain equivalent particle sizes. Each batch was mixed in a Universal Motor Drive 400 (Pharmag GmbH, Hamburg, Germany) attached to a cube mixer. The conditions for mixing all batches were kept the same at 50 RPM for 15 min. Premixed batches were fed to an MP19TC25 APV Baker 16 mm co-rotating twin screw extruder (APV Baker, Newcastle-under-Lyme, UK) equipped with a purpose-built filament for Ming dye. The filament dye has a conical shaped cavity that narrows it away from the extruder, which finishes in a circular orifice (diameter 2.30 mm). The gradient temperature profile of the HME process is detailed in Table 3. The screw speed was set at 80 RPM and the feeding rate was 0.4 kg/h. The extruded batches were hauled off by using a conveyor belt system consisting of a Teflon belt that was tilted at a 45° downward angle from the extruder die. A second conveyor twin-belt system was set at a sufficient haul-off speed to maintain a filament diameter of 1.75 ± 0.10 mm necessary for the FFF 3D printing process.

### 2.3. Production Tablets by FFF

**Figure 1:**
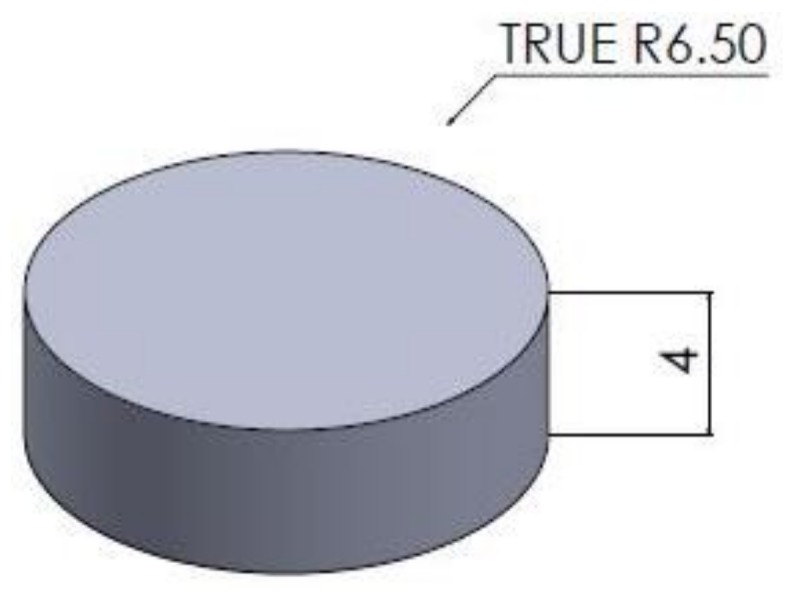
Three-dimensional design of a flat-face plain tablet.

FFF using F11 and formulations labelled “3DP” from Table 2 as feedstock material was carried out using a commercial desktop 3D printer, MakerBot Replicator 2× (Makerbot^®^ Industries, New York, NY, USA). The printing conditions for the most aesthetic and robust tablets were kept constant by using the following parameters: extrusion speed (10 mm/s), extruder temperature (150 °C), printing bed temperature (50 °C), extruder travel speed (50 mm/s), number of shells (1), roof and floor thickness (0.5 mm), layer height (0.2 mm), and infill 25% in which the linear infill pattern and the raft and support options were turned off. A total of 40 tablets were printed using these settings without any operator intervention after setup. The process was completed in 44 min. The three-dimensional design for a flat-face plain tablet was created using SolidWorks 2014 (Dassault Systèmes, Waltham, MA, USA) and saved as an STL extension format (see Figure 1). The STL file was opened using the monitor and remote control software suite MakerBot Desktop (Makerbot^®^ Industries, New York, NY, USA).

### 2.4. Mechanical Testing

#### 2.4.1. Dynamic Mechanical Analysis

Dynamic mechanical analysis (DMA) was performed on filaments of all formulations using TA Instruments DMA Q800 (Dublin, Ireland). The test was performed in single cantilever mode using a frequency of 1 Hz and an amplitude of 15 µm. The temperature range between −80 °C to 150 °C with 3 °C/min rate was used to determine the storage modulus, the loss modulus, and the glass transition temperature (tan δ) for all twelve formulations.

#### 2.4.2. Filament Stiffness

The temperature range −80 °C to 150 °C with 3 °C/min rate was used to determine the stiffness and glass transition temperature (tan δ) for PVP-VA, PCL, and the eleven formulations. The test was carried with a constant frequency of 1 Hz and an amplitude of 15 µm using the single cantilever mode. Equation (1) is the general equation of stiffness.

(1) $$Stiffness=\frac{Load}{Deformation}$$

Load is defined as the force applied to the material in any given moment to obtain the desired amplitude expressed in newtons (N). Deformation is the distance the sample has moved from its original position at the beginning of the test and it is expressed in meters (m).

#### 2.4.3. Filament Brittleness

The calculation of filament brittleness involved two separate tests, which were performed on 25 mm filament lengths of all formulations using a TA Instruments DMA Q800 (Dublin, Ireland). Storage modulus (E′) values were taken in single cantilever mode at room temperature with a frequency of 1 Hz. Cylindrical samples had a length of 17.5 mm and varying diameters. The test was performed in triplicate. Quasi-static 3-point bending of 25 mm filament lengths was performed separately on the Q800. The force applied to the samples was ramped up at 3 N/minute and the test was stopped when samples broke or a maximum displacement was achieved. The Brostow-Hagg Lobland-Narkis Equation (Equation (2)) for brittleness was used to obtain brittleness (B) values [37]. In the equation, E′ is the DMA storage modulus at 1.0 Hz at room temperature and strain-at-break (%), ε_b_ is calculated from room temperature 3-point bending.

(2) $$B=\frac{1}{\varepsilon_{b}\cdot E^{\prime }}$$

### 2.5. Melt Flow Indexing

Melt flow indexing (MFI) was performed for all formulations in a range of temperatures. The melt flow rates (MFR) were measured using a Zwick Roell Cflow extrusion plastometer, which was equipped with a 2 mm orifice die. All testing was performed following the guidelines of the ASTM standard D1238-13 with a fixed weight of 2.16 kg. Test temperatures were 140 °C and 150 °C based on the HME die temperature and FFF printing temperature, respectively.

### 2.6. Differential Scanning Calorimetry

Differential scanning calorimetry (DSC) was employed for thermal characterization of the virgin polymers and eleven formulations using a TA Instruments DSC 2920 Differential Scanning Calorimeter (Dublin, Ireland). Samples weighed between 8–12 mg and were placed in non-hermetical aluminum pans, which were crimped prior to testing with an empty crimped aluminum pan for reference. Each sample was summited to a heating cycle to remove thermal history consisting of a ramp from room temperature to 200 °C at a rate of 10 °C/min. A cooling cycle to 0 °C was set at a rate of 5 °C/min. Data recording was activated and the temperature was ramped up at a rate of 10 °C/min until it reached 200 °C.

### 2.7. Mass Loss Studies

Polymer filaments with a length of 20 mm were tested in duplicates in dissolution media of 0.2 M hydrochloric (HCl) acid, pH 1.2, and the temperature maintained at 37 ± 0.5 °C to mimic the stomach conditions during fasting. The stir rate was set to 60 RPM and 25 mL of dissolution media was used per vial for the filament strands and 50 mL for the tablets. At predetermined time intervals, samples were withdrawn from vessels, air dried, and weighed. Samples were then placed in the oven for 12 h at 40 °C and weighed again.

### 2.8. Direct Compression

Dried powder was mixed in a Universal Motor Drive (UMD) 400 (Pharmag GmbH, Klipphausen, Germany) that was attached to a cube mixer and rotated at 50 RPM for 15 min. The mixed batch was dried for a period of 12 h in an oven (Sanyo Gallenkamp, Loughborough, UK) at 40 ± 0.1 °C before being dry compressed for the manufacturing of compressed tablets. The tablet press used was a manual Atlas Series laboratory hydraulic press (Specac Limited, Orpington, UK) capable of 15 tons of pressure. The die was a hardened stainless steel evacu-able pellet die Specac GS03000 (Specac Limited) that produces tablets or disks with a diameter of 13 mm. Tablet formulations are in Table 2. About 500 mg of powder was accurately weighed on a Sartorius analytical balance (Sartorius, Weender Landstr, 94-10837075 Göttingen, Germany) and fed into the die. The dice and plunger were put on top of the powder and a 5-ton pressure was applied to the mixture for 30 s.

### 2.9. Drug Release Studies

Dissolution testing of direct compressed and 3D printed tablets was performed using a Distek dissolution system 2100B with a Distek temperature control system TCS 0200B (Distek Inc., North Brunswick, NJ, USA) using to USP Dissolution Apparatus I. The tablets were tested (*n* = 6) in dissolution media 0.2 M hydrochloric acid, pH 1.2, and the temperature maintained at 37 ± 0.5 °C to mimic the stomach conditions during fasting. The stir rate was set to 50 RPM with 900 mL of dissolution mediaused per vessel. At predetermined time intervals, 5 mL was withdrawn from each vessel and replaced with pre-heated media. The withdrawn samples were filtered through 0.45 µm filter and drug release determined at 272 nm by performing UV spectroscopy on a Shimadzu UV-1280 UV-VIS spectrophotometer (Shimadzu, Milton Keynes, UK), which was blanked with a solution of the buffer and dissolved polymers according to the formulation tested in order to secure the detection of caffeine. The dissolution profile was observed from a plot of time versus absorbance.

### 2.10. Scanning Electron Microscopy

Scanning electron microscopy (SEM) was performed on a Mira SEM (Tescan, Oxford Instruments, Cambridge, UK) using a range of magnifications to evaluate the surface morphology of samples through the secondary electrons function. Samples were placed in a petri dish and liquid nitrogen was poured into the dish with enough to completely submerge the samples in the liquid. The lid was placed on the petri dish and left until the nitrogen totally evaporated, which was immediately followed by the transversal break of samples. Afterward, the surface of the specimens and the cross-section were examined. First, the samples were placed on an aluminum stub and were gold coated using Baltec SCD 005 sputter coater (BAL-TEC GmbH D–58579, Schalksmühle, Germany) for 110 s at 0.1 mBar vacuum before observation.

### 2.11. Statistical Analysis

Data handling and analysis was performed using the Minitab 17 (Minitab Ltd., Coventry, UK). Test data was inputted into the software and, for replicate sets of data, mean and standard deviation values were calculated. The significance threshold was set at 0.05. The mean values were presented in the figures included in the results section and error bars represent standard deviation unless otherwise specified in the figure caption.

## 3. Results

### 3.1. Mechanical Characterization

#### 3.1.1. Filament Stiffness

**Figure 2:**
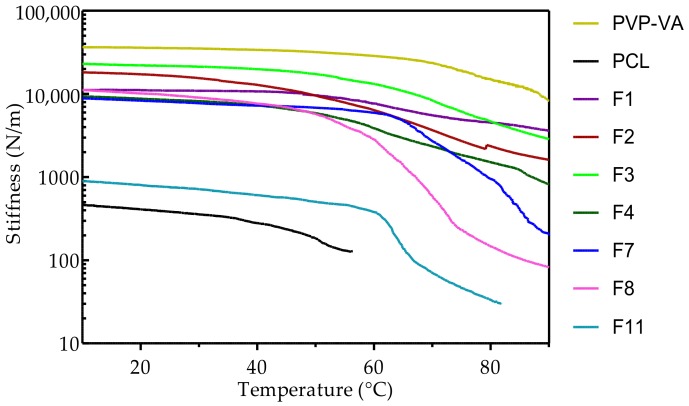
Stiffness (N/m) of extruded filaments within no manual working range for the FFF (fused-filament fabrication) process (10 °C to 90 °C) (*n* = 2).

Filament stiffness over no minimal FFF working range of a select number of melt-blended formulations are shown in Figure 2. The nominal working range reflects the temperature range the filament experiences as feed above the driving gear (room temperature) and as a piston below the driving gear (above 30 °C). Addition of 10% (*w*/*w*) of the recommended plasticizer P188 (F1) decreased PVP-VA stiffness at room temperature by 69%. Melt-blending with either PEO or PCL significantly decreased room temperature filament stiffness (*p* < 0.05). A 10% (*w*/*w*) addition of PEO (F3) decreased PVP-VA stiffness by just over 66%. PVP-VA stiffness decreased by 48% in addition of 10% (*w*/*w*) PCL (F2) and continued to decrease (75%) with double the amount of PCL (F4). Binary blends of higher amounts of PCL had no further effect on the PVP-VA room temperature filament stiffness. PVP-VA was over 200 times stiffer than PCL (306 N/m). Stiffness readings could not be made for PCL at temperatures above 56.05 °C since the polymer had started to melt. At higher piston temperatures, PVP-VA maintained a constant stiffness up until an inflection point (onset temperature) of 68.66 °C. Above this temperature, stiffness steadily decreased with increasing temperature. While higher PCL content did not significantly affect room temperature stiffness (*p* < 0.05), the higher the PCL content, the steeper the decline in stiffness was with rising temperature. For the final formulation (F11) addition of 10% (*w*/*w*), PEO significantly decreased the stiffness across the entire working temperature range. Above 60.0 °C, there was an abrupt drop-off in filament stiffness for the final F11 formulation. 

#### 3.1.2. Filament Brittleness

Table 4 shows the strain-at-break (ε_b_), storage modulus (E′), and brittleness B × 10^4^ values obtained from filaments of the different material formulations. The B values (%Pa) were derived from two separate mechanical tests, quasi-static 3-point bending that calculated ε_b_ values (%) and a room temperature dynamic mechanical test in a single cantilever (1 Hz) calculated the E′ values (Pa). PVP-VA showed the highest brittleness of polymers and blends with a value of 6.22 %Pa (10^4^) while PCL had a value that was 94.3% lower than that of PVP-VA at 0.35 %Pa (10^4^). The addition of 10% (*w*/*w*) P188 (F1) to PVP-VA decreased its strain-at-break along with an increase in brittleness by 34%. In contrast, 10% (*w*/*w*) PEO (F3) reduced the brittleness of PVP-VA by 66%. Doubling the amount of PEO (F5) further decreased the brittleness by an additional 4%. PCL decreased the overall brittleness of PVP-VA even though the effect was not as strong as that of PEO at the same concentrations. At 30% PCL (F6), brittleness values saw a reduction of 81%. F7 is composed of 40% (*w*/*w*) PCL and the lowest value of brittleness was observed at 0.10 %Pa (10^4^). Ternary blends (F9, F10, and F11) containing P188 or PEO in addition to PCL displayed similar low brittleness values of ~0.15 %Pa (10^4^).

#### 3.1.3. Dynamic Mechanical Analysis

**Figure 3:**
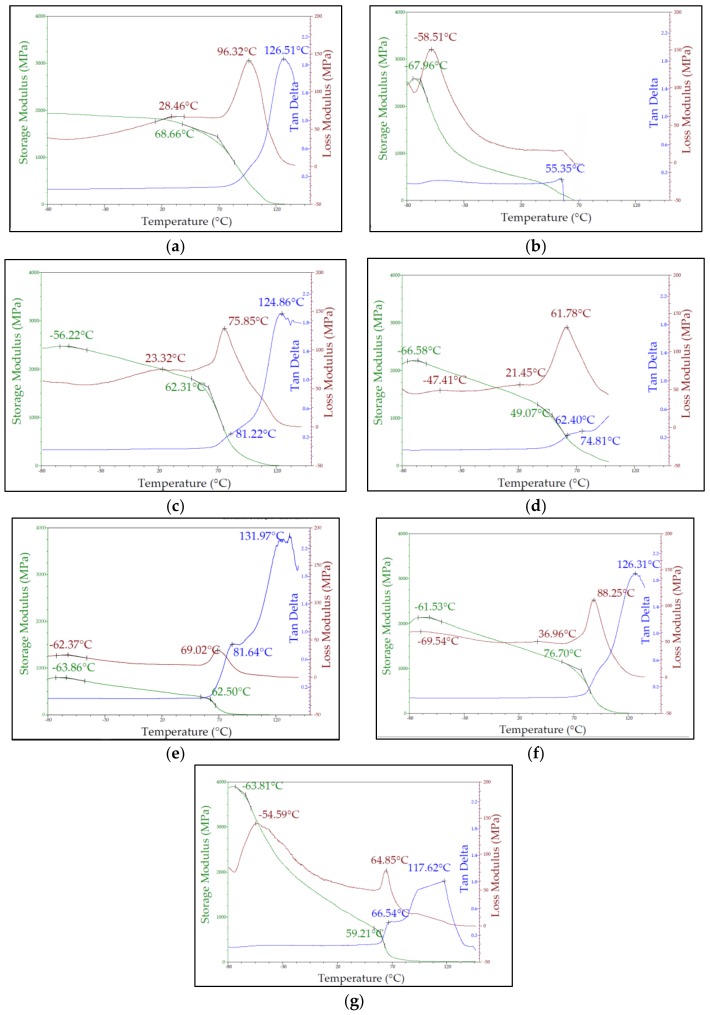
DMA (dynamic mechanical analysis) thermograms for a select number of formulations displaying storage modulus (E′), loss modulus (E″), and tan δ across a broad temperature (°C) sweep: (**a**) PVP-VA; (**b**) PCL; (**c**) F2; (**d**) F4; (**e**) F7; (**f**) F9, and (**g**) F11.

Figure 3 displays DMA thermograms for a selected number of formulations including showing the storage modulus (E′), loss modulus (E″), and tan δ across a broad temperature (°C) sweep. The storage modulus (E′) value for PVP-VA steadily decreased until the onset of a relaxation at 65.21 °C when E′ value declined more steeply (see Figure 3a). PCL had a storage modulus peak at −67.86 °C of 2479 MPa, which reflects the glass transition temperature (T_g_) and with increasing temperature E′ values steeply declined until around −45.00 °C when the rate of decline slowed before another sharp drop prior to melting (see Figure 3b). An addition of 10% (*w*/*w*) PCL to PVP-VA (see Figure 3c) produced a slight peak at −56.22 °C of 2463 MPa. This lower temperature peak increased in intensity and decreased in temperature with increasing PCL content. The inflection point in the storage modulus (onset temperature) decreased in temperature with increasing PCL content up until 20% (*w*/*w*) (see Figure 3c,d) 62.31 °C (F2), and 49.07 °C (F4). At 40% (*w*/*w*) PCL, the onset temperature rose to 62.50 °C (F7) and at 50% (*w*/*w*) PCL, the onset temperature rose further to 76.70 °C (F9). For the final formulation F11, which contained 10% (*w*/*w*) PEO, displayed a much steeper storage modulus decline after T_g_ (63.81 °C) and it should be noted that E′ value at T_g_ was significantly higher than for all the binary blends.

The loss modulus (E″) for PVP-VA displayed a sharp peak at 96.37 °C (see Figure 3a) while a sharp peak for PCL was at −59.06 °C (see Figure 3b). A secondary broad peak was apparent on the PVP-VA thermogram at 28.36 °C, which may be due to the VA comonomer. Addition of 10% (*w*/*w*) and 20% (*w*/*w*) PCL to PVP-VA reduced the temperature of the sharp peak to 75.98 °C and 61.31 °C and of the broad peak to 23.32 °C and 21.45 °C, respectively. For F4 (see Figure 3d), a second lower temperature broad peak appeared at −47.41 °C. For F7, with the addition of 40% (*w*/*w*) PCL (see Figure 3e), there was a significant decrease in the intensity of the sharp peak to 36.44 MPa and an increase in the temperature to 69.19 °C. A stronger lower temperature peak appeared at −61.91 °C and was sharper than previous low temperature broad peaks. At 50% PCL content (see Figure 3f), the sharp peak increased in temperature to 88.50 °C and two lower temperature peaks appeared at 36.96 °C and −69.54 °C. For the final formulation F11 (see Figure 3g), the higher temperature sharp peak was of low intensity and appeared at 64.11 °C. A distinct but rounded peak was present at −54.59 °C. 

Tan δ peaked at 126.68 °C for PVP-VA (see Figure 3a), but it is not clearly observed for PCL due to a noisy signal starting at 55.35 °C caused by the onset of melting. For F2, the peak at 121.69 °C corresponds to that of PVP-VA (see Figure 3c). There is no peak for F4 at the higher temperatures but a shouldered peak is observed around 74.16 °C (see Figure 3d). F7 displayed a more pronounced shouldered peak at 81.09 °C after which a crest formed at 131.97 °C (see Figure 3e). At 50% (*w*/*w*) PCL content, the shouldered peak was not observed but a strong, sharp peak was observed at 125.54 °C (see Figure 3f). The shouldered peak was present at 67.48 °C in the F11 sample, but the machine could not properly measure data points at higher temperatures (see Figure 3g).

### 3.2. Thermal Characterization

Table 5 displays the thermal properties of all the base polymers and the melt-blended formulations. Extruder torque is a measure of drive motor resistance due to melt-viscosity of the polymer inside the barrel [38] and it has been proposed as a measuring tool of the relative viscosities of polymer melts during the extrusion process [39]. During these studies, the temperatures and screw speed were kept constant for all formulations. Torque readings are shown in Table 5 for the different formulations. The highest torque reading was observed for PVP-VA while PCL was the lowest. Melt-blending PVP-VA with the other polymers reduced torque. Therefore, the resistance due to viscosity was reduced. The reduction of torque recorded during the extrusion process for mixtures of polymers when compared to PVP-VA allowed for higher manufacturing throughput. However, processing conditions were kept constant to guarantee all polymers were submitted to similar stresses during manufacturing. Both PEO and P188 have a plasticizing effect on PVP-VA, but this phenomenon was better observed with formulations containing PCL. Surging, which is due to inconsistencies in the amount of material pushed out the die, was conducted typically in a sinusoidal fashion and was soothed by the incorporation of PCL. The observed surging could be due to a number of factors such as material adhering to the screw, feed entry variations in the material particle shape, or inadequate filling of the metering section of the screw. Higher concentrations of PCL further reduced the inconsistencies of the extrudate geometry. The final formulation (F11) containing 10% (*w*/*w*) PEO was observed to have reduced instances of surging compared to the binary blends. 

Melt flow indexing is a simple test that measures the ability of a polymer to flow when in the molten state at a given temperature. The melt flow rates for polymers and blends are shown in Table 5 for both the temperature during HME (140 °C) and the established printing temperature (150 °C). PVA-VA had no melt flow at 140 °C along with PEO while P188 and PCL had the higher values of all polymer and formulations, 26.4 g/10 min, and 11.1 g/10 min, respectively. There is a direct correlation between the amount of PCL incorporated into PVP-VA and increasing MFR values with melt flow increasing from batches with 10% (*w*/*w*) PCL (F2) up to 50% (*w*/*w*) PCL (F8) by 4.1 g/10 min. PEO increased the melt flow of PVP-VA to a lesser degree than PCL. However, doubling its content from 10% to 20% (*w*/*w*) had the opposite effect by decreasing MFR values from 2.3 g/10 min to 1.8 g/10 min. F10 and F11 were found to possess the greater amount of melt flow with 9.3 g/10 min and 7.52 g/10 min, respectively. At 150 °C, PVP-VA flowed at a rate of 5.14 g/10 min, while PCL had an MFR of 17.23 g/10 min. Addition of 10% (*w*/*w*) of both P188 and PEO to the base polymer increased MFR of PVP-VA, with the P188 blend doubling the MFR. Addition of PCL to the PVP-VA increased the melt-flow rate. Adding 10% and 20% (*w*/*w*) PCL had a similar effect on melt-flow while increasing content up to 50% (*w*/*w*), which was shown to double the MFR. Ternary blends had differing effects on melt-flow depending on the third polymer. Adding 10% (*w*/*w*) PEO reduced MFR and adding 10% (*w*/*w*) P188 increased MFR. The final material formulation, which contained 60% (*w*/*w*) PCL and 10% (*w*/*w*) PEO, had an MFR of 10.53 g/10 min at 150 °C.

**Figure 4:**
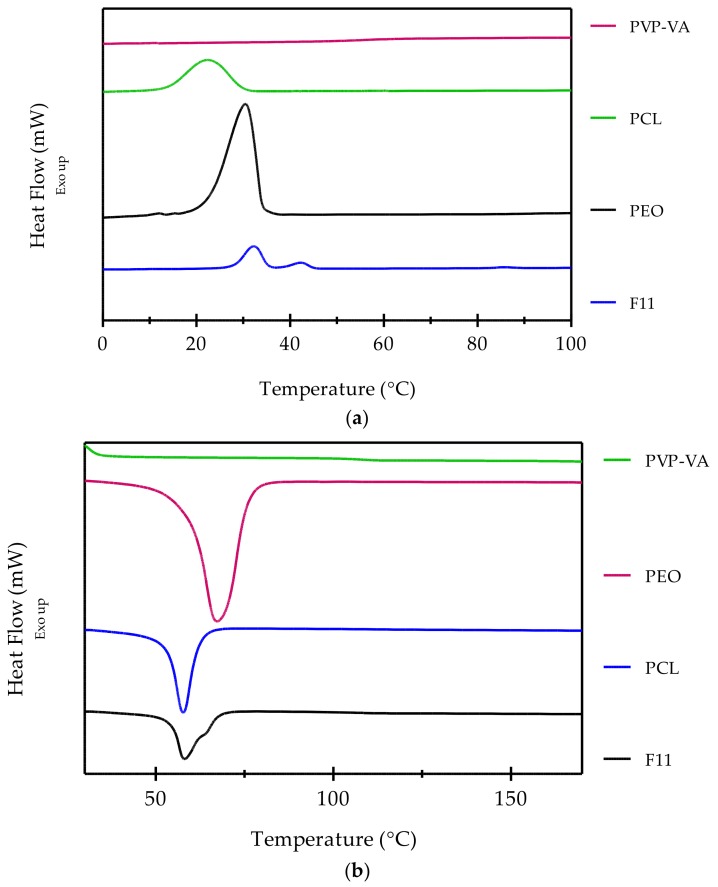
Overlaid DSC (differential scanning calorimetry) thermograms of the three base polymers and the F11 melt-blended formulation: (**a**) cooling and (**b**) heating.

Figure 4 shows the DSC thermograms of cooling (a) and heating (b) for PVP-VA, PCL, PEO, and the final formulation F11. PVP-VA is amorphous and thus did not generate a melting peak. The glass transition (T_g_) temperature of the polymer was 100.1 °C, which calculated from relaxations observed in DSC thermograms (Figure 4b) (*n* = 4) and is close to the peak observed at 96.32 °C in the loss modulus (see Figure 3a). A melting peak was observed at 57.9 °C for PCL while crystallization happened at 21.1 °C. PEO melted at 67.5 °C and solidified at 31.1 °C. The main melting peak for F11 occurred at 57.0 °C with a small shoulder at 62.2 °C. The main peak would represent the PCL portion of the ternary blend while the shoulder would represent the 10% (*w*/*w*) PEO portion. From the cooling cycles, two solidification peaks are observed at 30.4 °C and 43.2 °C, which would again represent PCL and PEO, respectively. A 5% (*w*/*w*) caffeine loading to F11 did not produce a melting peak at 235 °C to 237 °C and it did not alter the MFI values of the formulation. Therefore, further characterization is beyond the scope of this body of work (data not shown). 

### 3.3. Dissolution Studies

#### 3.3.1. Mass Loss

**Figure 5:**
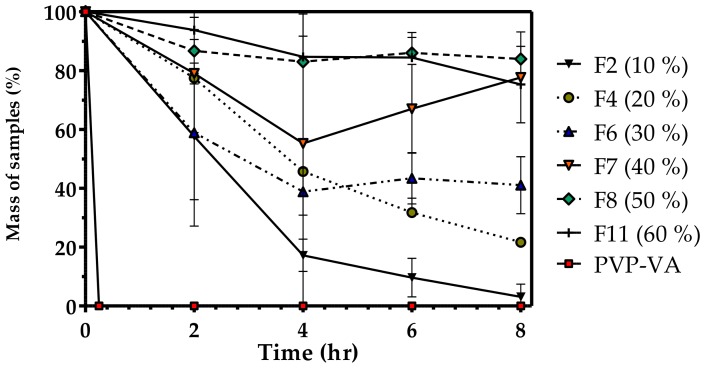
Percentage of mass loss in HCl media, pH 1.2, 0.2 M at different time points. Percentage values in legend correspond to PCL (polycaprolactone) content (*w*/*w* %).

The mass loss of select blends was measured over a period of 8 h to ascertain the effect of changes in material formulation on the disintegration profile of PVP-VA. Figure 5 shows the remaining mass (%) over time of a formulation filament in biologically relevant media. PVP-VA was shown to completely disintegrate within the first two hours, which would be expected for a polymer designed for immediate release applications. Adding PCL to the formulation slowed the rate of mass loss. Adding 10% (*w*/*w*) PCL reduced mass loss to 42.4% in the first 2 h by increasing to 82.8% at 4 h and only a tiny 3.1% portion remained after 8 h. Doubling the amount of PCL to 20% slowed mass loss even further, and after 4 h, more than twice the amount of mass remained (45.7%) compared to the 10% PCL sample. After 8 h, more than a fifth of the mass remained (21.7%) for this formulation. At higher PCL loadings, the linear pattern stopped. The sample containing 40% PCL is of particular note since it lost 44.8% mass after 4 h but regained 11.8% after 6 h. The formulations with a content of 50% (*w*/*w*) PCL and more displayed a slower rate of dissolution in media with more than 75% of their mass still intact after an 8 hour-period. 

#### 3.3.2. Cumulative Drug Release

**Figure 6:**
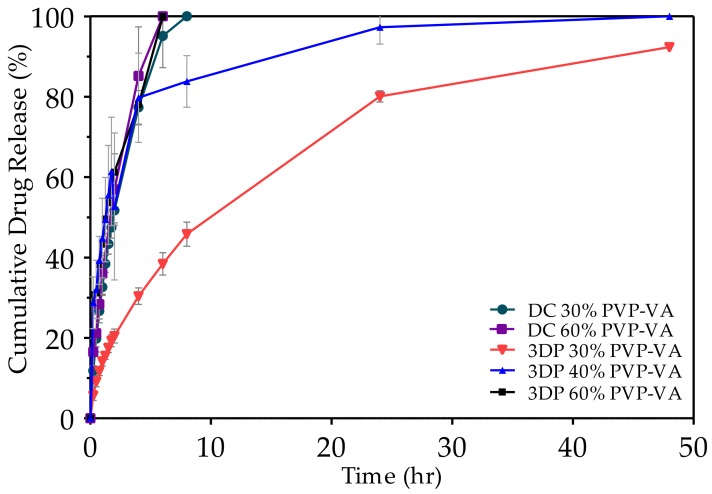
Cumulative caffeine release over 48 h in HCl 1.2 pH, 0.2 M media for different tablet formulations produced via direct compression. Percentage of PVP-VA (polyvinylpyrrolidone-vinyl acetate) reflects material composition only, which contains 10% *w*/*w* PEO (polyethylene oxide) with the remainder being composed of PCL. All formulations contain 5% *w*/*w* caffeine in the overall composition.

The influence of material formulation and tablet manufacturing processes on drug release over 48 h is shown in Figure 6. Two compressed tablets of different formulations (30% and 60% (*w*/*w*) PVP-VA content) both released over 75% drug content after 6 h. Similarly, a 3D printed tablet containing 30% (*w*/*w*) PVP-VA released 78.3% drug after 6 h. There was no significant difference in the release profiles of these three tablets (*p* < 0.05). The presence of PCL in the formulation slows caffeine drug release and the immediate release properties of PVP-VA. A PVP-VA compressed tablet released 100% drug in under 1 h (data not shown). For a 40% (*w*/*w*) PVP-VA 3D printed tablet, the cumulative release was not significantly different for time points up to 6 h. Beyond this point, the cumulative release from the tablet slowed significantly compared to other tablets. After 8 h and 24 h, the tablet had released 83.8% and 97.3% drug, respectively. There was a significant difference in the release profile of the 30% (*w*/*w*) PVP-VA 3D printed tablet and the other tablets at almost all time points. After 6 h, this tablet released 38.5% of the drug with 50.1% release of the 30% (*w*/*w*) PVP-VA 3D printed tablet. After 24-h release increased to 80.1% and, after 48 h, the release was 92.3%.

## 4. Discussion

### 4.1. Material Formulation Rationale

PVP-VA is a copolymer of polyvinylpyrrolidone (PVP) and vinyl acetate (VA). The addition of the VA side chains increases the hydrophobicity of PVP. The polymer has previously been used in the production of amorphous solid dispersions [40,41,42,43,44], as a release modifier [45], and has been blended with PCL for the production of tissue engineering scaffolds [46,47]. Our initial HME trials to produce PVP-VA filaments for FFF were unsuccessful. The material proved to be brittle and the filament would snap during the HME downstream haul-off process. Therefore, our approach was to modify PVP-VA sufficiently through melt-blending so that it would form a suitable filament. We aimed to find a material formulation incorporating PVP-VA, which would permit the production of a complete batch (*n* = 40) of flat-face plain tablets during a single print run. Kolliphor^®^ P188 is the recommended plasticizer by the supplier BASF^®^ [34]. In addition to the plasticizer, there were no observable changes in the flexibility of the filament. Very high loadings of P188 produced extrudate that would crumb and not form consistent filaments. Therefore, other polymers were investigated to blend with PVP-VA.

Blending during the HME process is a means of combining properties of different polymers into a single final object [48]. Melt-blending is not a new concept in drug delivery as with industrial applications. It is a means that provides the final dosage form with refined or a broader set of properties. We have previously reported on blending polyethylene vinyl acetate (PEVA) with polylactic acid (PLA) to improve the release of hydrophilic tenofovir from PEVA intravaginal rings [49]. The production of solid dispersions has benefited greatly from melt-blending. Polyethylene glycol (PEG) is by far the most widely used polymer in the production of solid dispersions due to low melting points, fast solidification behavior, and low toxicity [50]. However, such formulations made from the polymer are unstable. Some authors have described the positive impact of melt blending [40,50,51]. Bley et al. [40] describe the production of solid dispersions of PEG and different polymers via co-melting. The addition of polymers was aimed at stabilizing amorphous forms of water-insoluble drugs in PEG-based solid dispersions. The researchers found that blends of PEG with PVP-VA were less viscous than the pure polymers and that the PEG/PVP-VA blend created the best solid dispersion regarding both the dissolution rate and amorphous drug stability for both drugs. Therefore, melt-blending and careful polymer selection provided an advantage compared to using a single polymer.

Melt-blending for FFF has been described a number of times in prior studies. Rocha et al. [52] described the production filaments from acrylonitrile butadiene styrene (ABS) based binary and ternary polymer blends. The printed parts produced from the blends displayed different mechanical, physical, and surface properties compared to the neat ABS samples. Printability could be maintained across a broad range of compositions and miscibilities. Roberson et al. [53] described the utilization of melt-blending to develop materials for specific applications and how it can be used to overcome specific shortcomings inherent to printing with the neat polymers. The same group described melt-blending ABS with thermoplastic elastomer styrene ethylene butylene styrene (SEBS) grafted with maleic anhydride to produce a flexible material suitable for the production of actuators [54]. Through melt-blending, the authors were able to produce prints with comparable performance to those using higher cost polyurethane filaments. Although the majority of other studies are concerned with non-pharmaceutical polymers [55,56,57], some researchers are examining melt-blends in FFF for medical applications. Kosorn et al. [58] produced blends containing different compositions of polycaprolactone (PCL) and poly(3-hydroxybutyrate-*co*-3-hydroxyvalerate) (PHBHV) for porous scaffolds. Higher PHBHV content improved compressive strength, increased chondrocyte proliferative capacity, and enhanced chondrogenic potential. Alhijjaj et al. [26] used melt-blending to improve printability and control drug release from printed solid dispersions. The researchers created Eudragit EPO or Soluplus based blends with PEG, PEO, and Tween 80 and achieved excellent printability and drug dispersion. Blend composition had a significant influence over disintegration behavior and rates of drug release. 

Since PVP-VA proved to be unprintable due to brittleness and high stiffness, our strategy was to melt-blend PVP-VA with another polymer that had the inherent flexibility and ductility. Ideally, the polymer would also be well-established for FFF 3DP, drug delivery, and be biocompatible. One polymer that fits the criteria is PCL. The polymer has a long history in the FFF 3DP and one of the earliest research articles on FFF for biomedical applications described the use of PCL in the production of a scaffold [59]. PCL-based drug delivery systems present high drug permeability, excellent compatibility with many drugs, and full excretion from the body once absorbed, which makes the polymer an excellent choice [60]. A possible disadvantage of PCL is the slow degradation rate that would likely impede the immediate release properties of PVP-VA, but this was not a hindrance for us since our main consideration was to use a material that could be utilized in HME, direct compaction, and injection molding. For the interested reader, a recent paper by Solanki et al. [61] describes a formulation strategy for FFF that maintains the immediate release properties of PVP-VA through melt-blending with hydroxypropyl methylcellulose and hydroxypropyl methylcellulose acetate succinate. PEO was also included in the current formulation trials since we wished to reduce the hydrophobicity of the PCL [62]. We have previously had success blending PCL with PEO to form an oral tablet [38] and such blends have been reported elsewhere as efficient drug carriers [63,64,65]. 

### 4.2. Filament Production

**Figure 7:**
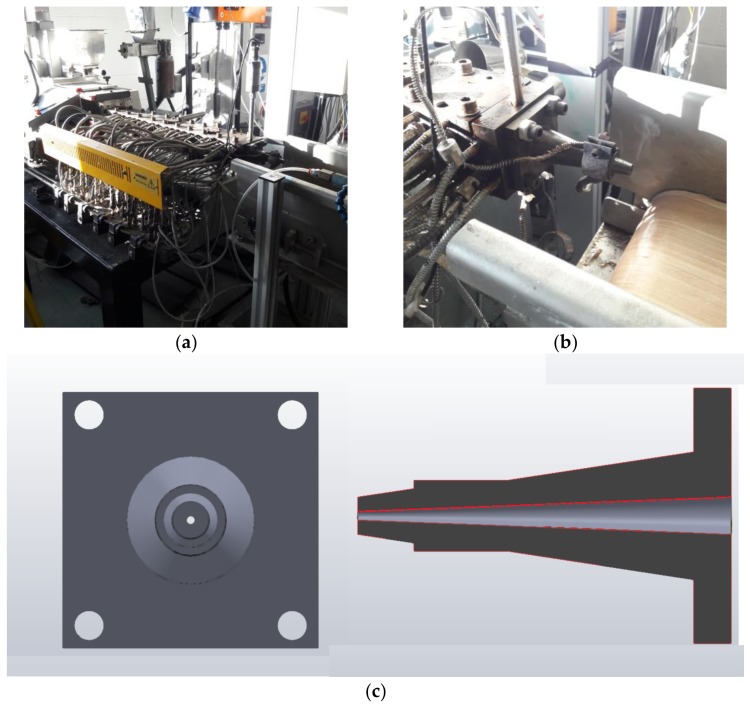
Depiction of machinery used for the fabrication of formulations described in this body of work: (**a**) twin-screw extruder, (**b**) mounted die attachment on extruder flange, and (**c**) schematic of die attachment.

For our purposes, we wished to melt-blend polymers with drug and create filament in a single step. To do this requires the use of a twin-screw extruder that provides for better mixing of the drug within a polymer compared to a single screw extruder [30]. Figure 7a,b show our filament extrusion setup and Figure 7c is the design of the die attachment that was attached to the front-face of the twin-screw extruder. The conical design for the attachment allows for an increase in die pressure without applying excessive shear force on the polymer melt since excessive shear can degrade certain polymers [66]. The design also permits for a steady flow of material out of the extruder. The consistency is a key feature needed for the manufacture of FFF filament strands since the margin of tolerance for the dimensions of the extrudates is narrow. The strands needed to have a diameter of 1.75 mm ± 0.10 mm to pass through the driving gear and into the liquefier. Any values below or above this range are not feasible as a feedstock material for the MakerBot^®^ 3D printer.

**Figure 8:**
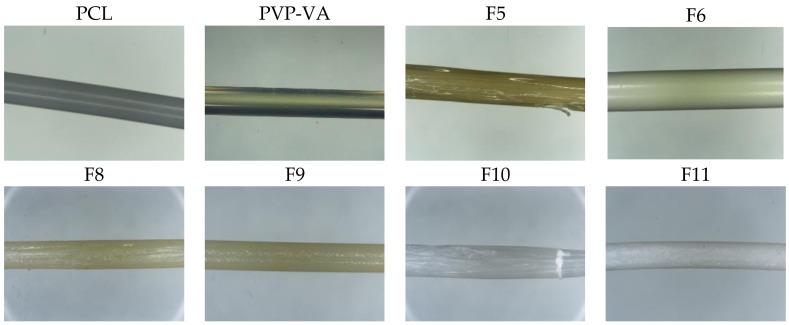
Physical appearance of filaments from select formulations made via hot-melt extrusion.

The front orifice of the die attachment was designed with a diameter of 2.30 mm, which allowed the extruded filament to be larger than needed so that control of the filament diameter was completed through subsequent unidirectional stretching by the haul-off units. Since we did not have access to a melt-pump system, compensation for extrusion surging was through operator control of the haul-off speed. The extrudate filament was cooled through a system of air knives and not through a water-bath to prevent erosion of the water-soluble polymer filament and drug loss. The haul-off system was in two stages: a first Teflon belt at a 45° decline with air knife cooling and a second twin belt conveyor that was the dominant haul-off controller. Figure 8 shows the physical appearance of a select number of filaments produced during HME trials. The majority of formulations gave filaments with a rough surface, which is indicative of the onset of sharkskin. The sharkskin appearance is indicative of instabilities in the flow exiting the die [67] and is probably related to the immiscible portions of the melting portion. Higher die temperatures may have resolved the issue. Both polymers, PVP-VA and PCL, and F6 all produced filaments with a smooth surface with no sign of sharkskin, which suggested stable melts at these processing temperatures [67]. For the most part, the die attachment reduced surging from the twin-screw extruder, but some operator intervention was still required to maintain tolerances. Addition of 10% (*w*/*w*) PEO had the unexpected benefit of almost eliminating extrusion surging in the final 60% (*w*/*w*) PCL formulation.

### 4.3. Filament Characterization

For PVP-VA printability to improve, it had to be modified to remove brittleness and decrease stiffness. Figure 2 shows stiffness of the filaments for a select number of formulations. On melt-blending, with the other polymers, the stiffness was reduced sufficiently to permit coiling. We estimate from our data that filament stiffness should not exceed 1000 N/m to enable consistent coiling. In addition, from our experience, as stiffness surpasses 10,000 N/m, the printable length of filament shortens. Filaments must also be able to resist buckling after the driving gear due to the force applied during feeding since the filament acts as a piston on the molten polymer in the liquefier [33,68]. Venkataraman et al. [68] derived a relationship of elastic modulus (in compression) to apparent viscosity in which a critical ratio (3.00–5.00 × 10^5^ s^−1^) ensures a material will not buckle during FFF, i.e., the filament is sufficiently stiff to act as a piston and drive out molten polymer in the liquefier through the nozzle. Insufficient stiffness was not an issue for PVP-VA and the reverse was more of a concern since the filament could not be coiled for proper feeding. The column strength critical ratio to prevent buckling can be assumed to have been maintained since buckling was not observed. We were unable to directly calculate the ratio without access to a capillary rheometer.

The second main issue with PVP-VA was the inherent brittleness that created issues during filament production and feeding of the FFF extrusion head. To quantify brittleness, the Brostow-Hagg Lobland-Narkis equation for Brittleness (B) (Equation (2)) was used [37]. Strain-at-break (ε_b_) is a measure of a material’s ductility and is usually recorded during tensile testing as elongation-at-break (%). Storage modulus (E′) is the solid-like (elastic) response to stress and is usually recorded during dynamic mechanical analysis (DMA). The authors specify the DMA conditions at room temperature for a frequency of 1 Hz. The elegance of this equation is that it requires results from two forms of mechanical testing—quasi-static and dynamic. For convenience, we took results directly from extruded filaments and not tensile specimens. Therefore, we derived brittleness (B) from strain-at-break (ε_b_) values obtained from 3-point bend testing by using deflection rather than elongation values. The true value of this approach derives from direct testing of filaments with comparable stress (flexural) applied to filament passing through the driving gear system. The equation should enable researchers to prescreen material formulations for suitability. Since the driving gear mechanisms of different FFF printers will vary, researchers can determine the critical brittleness (B_c_) for their system, above which it will be known that the filament will fail. The results in Table 4 aided us in quantifying observations about filaments that had failed to print since the filaments that had failed to negotiate the Makerbot^®^ system had B values higher than 2.00 %Pa (10^4^). Thus, B < 0.0002 %Pa will be a critical material characteristic for future material formulations for this printer. 

**Figure 9:**
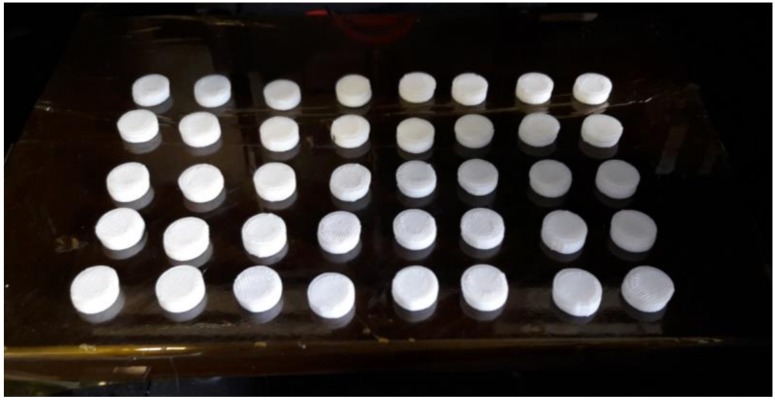
Complete batch of flat-faced tablets produced via FFF 3D printing. Total of 40 tablets covered the print bed of MakerBot Replicator 2X.

Figure 9 shows the finished print of a complete batch of tablets. Part of the pre-screening process is to calculate the length of filament required to print a complete batch of 13 mm diameter tablets. Most printer software will pause a print mid-run to permit changing of the filament and, therefore, it is not an insurmountable issue, but from a purely practical point-of-view, it is important to be aware of how large of a batch can be printed from a single filament length. Equation (3) was used to calculate the density (D_b_) of the ternary blend F11 (0.001141 g/mm^3^). The total volume (V_T_) of forty 4 mm high (cylinder) tablets was 21,237.16 mm^3^ (Equation (4)) where $h_{T_{s}}$ is the total height of sample tablets combined and r_s_ is the radius of the tablet. The total mass (M_T_) to print forty 100% infill tablets was calculated at 24.23 g (Equation (5)). Finally, the length of required 1.75 mm diameter filament to print forty 100% infill tablets could be calculated as 8829.32 mm using Equation (6) where r_f_ is the radius of the filament. We refer to this as the minimum batch length (L_B_). The filament length should be adjusted for the percentage infill (x) of samples, e.g., 0.25 for a 25% infill, which reduces the length to 2207.33 mm. In addition, to account for variation in filament diameter and the material needed for the outer shell, a correction factor of at least 1.3 should be applied, which would bring the minimum batch length (L_B_) for these tablets to 2869.53 mm. In further criteria, we set any material formulation is the minimum sample length (L_S_), which is the minimum filament length to print a single sample without operator intervention. The L_S_ for this design of flat-face tablet with 25% infill is 71.74 mm. We consider L_S_ to be the minium criteria of viability for any material formulation for the FFF process. PVP-VA could not pass this minimum criteria (L_S_) due to brittleness. F5 was the first formulation to pass this criteria, but only F11 could succeed in passing the minium batch length (L_B_) and provide a filament in excess of 8.83 m. To achieve the L_B_ required for the quite high PCL content, it ultimately reduced the drug release rate (see Figure 6). Therefore, printability versus the drug release profile is a choice that can guide future formulation. We can maintain more of the immediate release properties of PVP-VA by reducing PCL content but at the cost of the filament length and subsequent batch size. L_s_ is the limit at which PCL content can be reduced.

(3) $$D_{b}=x_{1}D_{1}+x_{2}D_{2}+x_{3}D_{3}$$

(4) $$V_{T}={\pi h}_{T_{s}}r_{s}^{2}$$

(5) $$M_{T}=D_{b}V_{T}$$

(6) $$L_{B}=\frac{h_{T_{s}}r_{s}^{2}}{r_{f}^{2}}$$

### 4.4. 3D Printing of Flat-Faced Tablets

Formulation F11 was chosen for the material blend’s ability to overcome the physical restrictions of the FFF process and to form a consistent filament to be fed to an extrusion head. Filament is usually spooled at the point of production on the downstream equipment. Spooling is usually the most convenient approach with spools sold in ~1 kg batches. However, it is possible to create successful prints with unspooled filaments if it is unobstructed and can move freely. Figure 9 shows the finished print of a complete batch of tablets. Forty 13 mm diameter tablets is the maximum number that could be consistently printed on the print bed of the Makerbot^®^ system. The print-bed is covered with a disposable high-temperature Kapton^®^ polyimide tape, which glues the first layer deposited. No raft or support structures are required for flat-face tablets. The outer wall of tablets was made by one solid shell. The roof and floor were also solid and had a depth of 0.5 mm. Infill density was set to 25% and the infill pattern was linear. These settings create a tablet with a shell structure with 75% of its inner volume consisting of void space.

**Figure 10:**
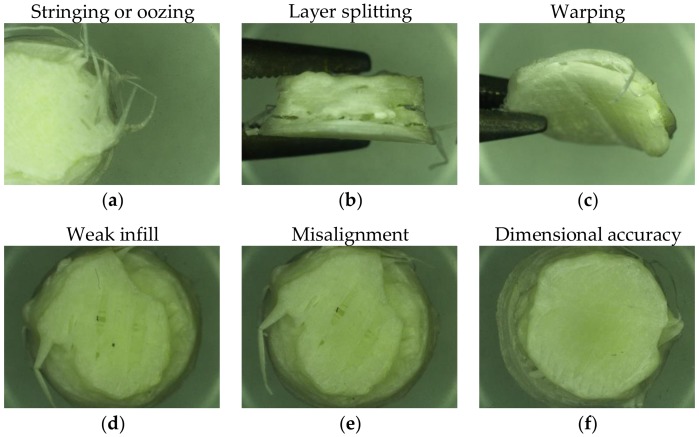
Most common print deformities that occur during the FFF 3D printing.

Figure 10 shows examples of the main types of part failure during the FFF process. Stringing (see Figure 10a) occurs when excess material on the nozzle is dragged from the part during manufacturing. The problem is more pronounced for materials with a high melt strength that will readily allow for stretching of molten beads. Some printing software has a ‘retraction’ countermeasure setting that eases back pressure in the extrusion head to prevent oozing from the nozzle during print head travel. Other reasons that could cause this flaw are two high nozzle temperatures that cause low material viscosity over extrusion of material and slow cooling of deposited material due to high print bed temperatures. Layer splitting (see Figure 10b) occurs due to inadequate layer coalescence during deposition. The polymer chains in the depositing molten layer must intermingle with the polymer chain of the previous layer to achieve proper coalescence. If adequate coalescence is not achieved, then during cooling, elimination will occur and layers will split apart. Higher printing and print-bed temperatures will overcome this issue since lower viscosities and softening of printed layers will both promote coalescence. Warping (see Figure 10c) is a phenomenon that is not restricted to FFF but occurs in other processes including injection molding [69]. In the FFF process, it occurs due to poor adhesion of the base layer to the print-bed and when subsequent layers are deposited on top. Internal stress between the layers of the print causes the part to warp and curl away from the print-bed surface. One of the main reasons for warping is the too low print-bed temperature that creates an excessive thermal gradient [70].

Infill determines the amount of material printed between the outer shells of a 3DP part. A weak infill (see Figure 10d) will fail to provide inner support to the part, which compromises the final mechanical integrity. Weak infill can be caused by choosing the wrong infill pattern for the specific inner geometry of the part. Too high a printing speed that prevents consistent layer deposition and poor layer deposition as a result of inconsistent feeding due to problems with the feedstock or melt-feed. Misalignment (Figure 10e) is due to discrepancies in the printers and the x-y-z axis dimensions. Most FFF printers have an open loop system without feedback sensors, which means that the printer will print the preprogrammed CAD design regardless of any misprint in the previously deposited layers. Assuming the print-bed is properly calibrated, the operator must manually adjust program settings based on the performance of the material to ensure that the settings (print speed, layer height, layer thickness, and more) are achievable. Other reasons for misalignment are related to hardware issues such as deficiencies in the stepper motor or tension belts. Relatedly, dimensional accuracy (see Figure 10f) is caused by extrusion problems, print-bed calibration accuracy, and filament quality. Any fluctuation in the material being deposited will disrupt the dimensions of the part while a nozzle that is closer or further than intended from the printing bed will have a similar consequence to the former.

### 4.5. Tablet Properties

Mass loss and drug release studies were used to assess the effect of changing material formulation for dissolution. As would be expected, adding a hydrophobic PCL to the PVP-VA had a significant retardation effect on the mass loss rate (see Figure 5). The PCL content significantly reduced the drug release rate (see Figure 6) while PVP-VA fully released drugs within the first hour. The 60% (*w*/*w*) PCL took over 8 h to complete. For us, the immediate drug release properties of PVP-VA was not a critical factor for our tablets and was chosen for the suitability for both direct compression and HME tablet production processes. If immediate drug release had been a critical factor, then our material formulation could easily have been changed to suit this criterion. For example, the PEO content could have increased or we could have chosen a water-soluble polymer such as polyvinyl alcohol instead of PCL. Melt-blending has the inherent flexibility to change formulation at will to meet such needs. The different nature of the manufacturing processes influenced the release rate of the drug substance of tablets with the same formulations (see Figure 6). The HME process intimately mixes polymer chains in the molten state and they remain entangled when solidified. DC tablets contain the polymers as powdered mixtures that form strong interparticulate bonds during compression, but the polymer chains are not entangled. In addition, the DC tablet containing 60% (*w*/*w*) PCL completely disintegrated after 8 h, which enabled the total release of the drug.

**Figure 11:**
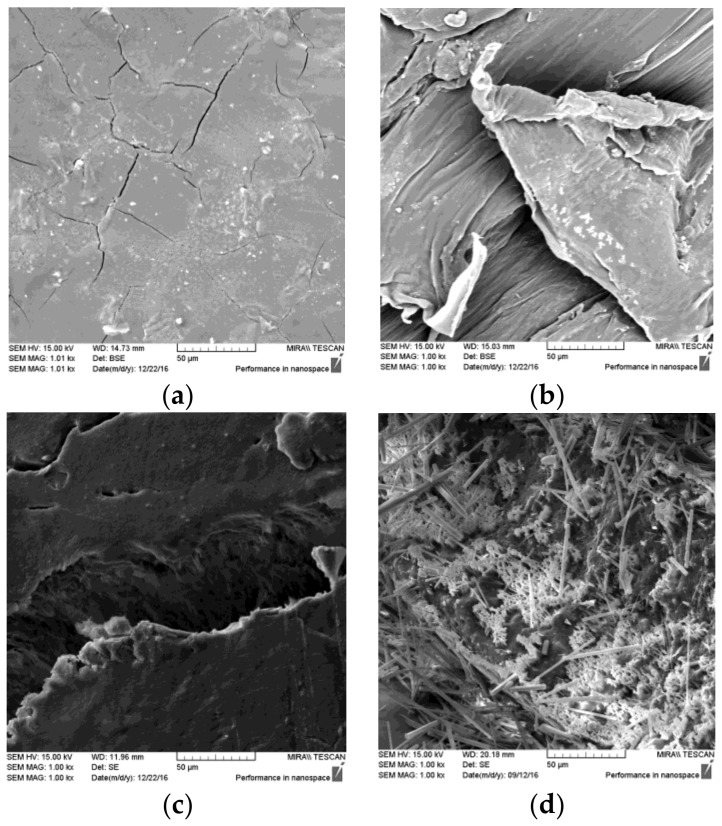
SEM scans of the three polymers and the final ternary blend containing 5% (*w*/*w*) caffeine: (**a**) PVP-VA filament cross-section; (**b**) PCL filament cross-section; (**c**) PEO filament cross-section; and (**d**) 25% infill 3DP tablet cross-section of F11.

The DMA thermograms show that the binary blends of PVP-VA and PCL are only partially miscible. Complete miscibility of binary blends usually coincides with the formation of a single T_g_ peak [71]. Tan δ peaks for the binary blends show that increasing PCL content produced two distinct T_g_ peaks, but these peaks moved closer together as PCL content increased. This is characteristic of partial miscibility [72]. The absence of two distinct Tan δ peaks with increasing PCL up to 20% (*w*/*w*) PCL and the appearance of two distinct peaks at higher loadings would suggest that PCL is miscible in PVP-VA up to 20% (*w*/*w*) content. Mass loss and drug dissolution data both suggest that when the PCL exceeds 20% (*w*/*w*) of the composition, the PVP-VA becomes entrapped with the PCL matrix as domains. Figure 11 shows SEM scans of the polymers and formulation F11 (containing 5% (*w*/*w*) caffeine). The increase in mass after 4 h during the mass loss study for the F6 and F7 blends could be due to the swelling of the PVP-VA domains encapsulated by the PCL matrix from ingress of media. SEM of the printed F11 tablet was inconclusive with regard to miscibility, other than showing that the morphology of the ternary blend was highly disordered. The open structure of 25% infill tablets is very clear. Monoclinic caffeine is clearly distinguishable and a white spongy layer is also visible. Since PVP-VA is glassy, it is more likely that this spongy layer is a PEO domain. The presence of PVP-VA is not readily discernible. Since miscibility was unimportant to us, it was not studied beyond the scope of the data presented and would warrant much deeper investigation to pick apart the miscibilities present within the ternary blend. Miscibility is only a criterion for FFF of solid dosage forms if immiscibility is detrimental to the performance of the filament or significantly impairs the performance of the final dosage form. In this study, as with others [52], any blend immiscibility did not impede the printing of parts.

### 4.6. Material Considerations

**Figure 12:**
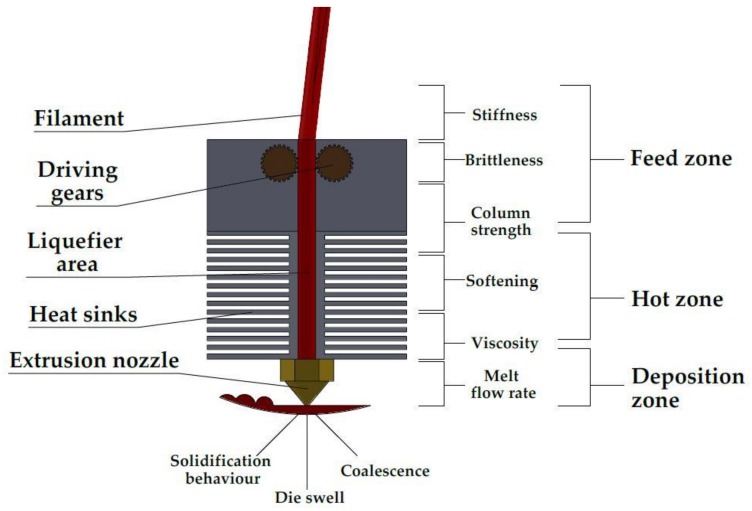
Detailed view of an FFF printer extrusion head with parts identified. The three distinct zones of the process are labelled and nine of the main material considerations are listed beside the sections of the extruder head in which they exert the most influence.

Figure 12 is a detailed schematic of an FFF extrusion head. It is best to consider the material in relation to each of the three zones of the FFF process—feed, heat, and deposition—since each zone has a specific set of challenges. The feed zone is governed by the bulk properties of the filament, which entails how successfully it copes with the driving gear mechanism. For the MakerBot^®^ printer, the driving gear system is part of the extrusion head assembly and, therefore, feeds directly into the liquefier. These are known as direct drive extruders. On other FFF printers, the driving gear mechanism on the side of the printer at a distance removed from the extrusion head is known as a Bowden extruder. The filament is driven along feed tubing to the extrusion head. Such a system severely restricts the material that can be printed since the filament has to be sufficiently flexible and aqueous to navigate the feeding tube. Our recommendation to other researchers who are producing filaments via heat-melting extrusion is only to purchase FFF printers that have the direct driving gear feeding system since it provides much-valued leeway for printing compared to a Bowden system. 

The heating zone is dominated by the material’s response to being heated in a chamber. A suitable material should be able to form a consistent melting factor in the most efficient manner. Innovation in this section is related to the heating elements by providing uniform, stable heat flux and eliminating hot-spots and dead zones so that the length of the liquefier is consistently heated. For the deposition zone, the material properties are dominated by the behavior of the material to flowing, cooling, and the ability to adhere to the previous layer or print-bed. Nozzle improvement aims through innovative design to eliminate or reduce known problems in layer deposition such as die swell and to improve print resolution. It is important to note that advances in driving gear and extruder head technology is more than an annual occurrence and existing printers can be retrofitted in most instances with extrusion heads that will accommodate a wider range of materials than what was previously the case. Advances aim to reduce extrusion head weight, increase reliability and repeatability, improve print resolution, and expand the range of materials that can be printed consistently such as softer thermoplastics. Table 6 is a compilation of the critical material properties that must be considered when approaching the production of solid dosage forms via fused-filament fabrication.

## 5. Conclusions

Fused filament fabrication is an HME-based 3D printing process that is finding increasing utility in pharmaceutical applications. However, the ready-use of established matrix polymers is limited due to the physical restrictions imposed by the mechanics of the process. We have described in detail the main considerations to be undertaken at each of the three zones of the standard fused-filament fabrication printers. We have also described an HME melt-blending approach that can be readily adopted by others for the production of solid dosage forms. Melt-blending is a well-established, cost-effective, and convenient means of combining the properties of two or more polymers into a single matrix material. The final properties of the matrix material can be altered by changing the composition of the polymers. For the formulation scientist, the melt-blending framework is suitably flexible to accommodate both the requirements of the final dosage form and any physical shortcomings of the main matrix polymer under evaluation during fused-filament fabrication.

## Figures and Tables

**Table 1:** Material formulations of melt-blends used in pre-screening.

Name	Composition by Weight (%)			
	PVP-VA	P188	PCL	PEO
PVP-VA	100	-	-	-
PCL	-	-	100	-
F1	90	10	-	-
F2	90	-	10	-
F3	90	-	-	10
F4	80	-	20	-
F5	80	-	-	20
F6	70	-	30	-
F7	60	-	40	-
F8	50	-	50	-
F9	60	-	30	10
F10	60	10	30	-
F11	30	-	60	10

**Table 2:** Material formulations for drug dissolution studies.

Name	Composition by Weight (%)			
	PVP-VA	PCL	PEO	Caffeine
DC 30% PVP-VA	30	55	10	5
DC 60% PVP-VA	60	25	10	5
3DP 30% PVP-VA	30	55	10	5
3DP 40% PVP-VA	40	−45	10	5
3DP 60% PVP-VA	60	25	10	5

**Table 3:** Temperature profile for twin-screw compounding HME (hot-melt extrusion) process to produce filament.

Temperature (°C)							
Zone 1	Zone 2	Zone 3	Zone 4	Zone 5	Zone 6	Flange	Die
80	90	100	110	120	130	140	140

**Table 4:** Brittleness (B) (%Pa) of extruded filaments at room temperature. B values are shown as multiples of 10^4^ for the convenience of the reader. Storage modulus (E′) was obtained at room temperature at a 1 Hz frequency (*n* = 3). Strain-at-break (ε_b_) was obtained using a room temperature three-point bend testing (*n* = 5).

Formulation	B (%Pa) (10^4^)	ε_b_ (%)	E′ (Pa)
PVP-VA	6.22	0.85 ± 0.19	1897.89 ± 2.27
PCL	0.35	59.07 ± 1.38	481.99 ± 0.04
F1	8.33	0.68 ± 0.08	1768.03 ± 61.47
F2	5.75	0.93 ± 0.12	1877.50 ± 19.19
F3	2.10	2.34 ± 0.85	2033.35 ± 24.26
F4	3.24	2.41 ± 0.67	1277.84 ± 2.76
F5	1.89	2.29 ± 0.82	2314.50 ± 6.26
F6	1.21	3.73 ± 2.28	2223.50 ± 59.54
F7	0.10	78.58 ± 5.65	1295.80 ± 305.20
F8	0.62	13.82 ± 5.34	1175.02 ± 34.18
F9	0.15	54.46 ± 30.79	1223.47 ± 1.55
F10	0.15	73.06 ± 4.15	935.16 ± 1.08
F11	0.14	72.23 ± 6.67	995.94 ± 1.87

**Table 5:** Extruder torque measurements, melt flow rates of polymers, and melt-blend formulations. Extruder torque measurements were recorded during twin-screw hot-melt extrusion compounding and are a measure of melt viscosity.

Name	Extruder Torque	Melt Flow Rate at 140 °C	Melt Flow Rate at 150 °C
	(%)	(g/10 min)	(g/10 min)
PVP-VA	40	0.00 ± 0.00	5.14 ± 0.12
PCL	10	11.10 ± 0.04	17.23 ± 0.77
F1	15	4.51 ± 0.04	9.51 ± 0.17
F2	20	3.01 ± 0.03	4.65 ± 0.70
F3	15	2.33 ± 0.03	3.12 ± 0.30
F4	20	2.89 ± 0.15	12.42 ± 0.41
F5	15	0.55 ± 0.01	1.8 ± 0.01
F6	15	4.74 ± 0.06	5.88 ± 0.15
F7	10	6.88 ± 0.07	8.37 ± 0.04
F8	10	7.06 ± 0.07	7.24 ± 0.05
F9	10	3.56 ± 0.05	6.93 ± 0.07
F10	10	9.30 ± 0.11	22.87 ± 0.69
F11	10	7.52 ± 0.06	10.53 ± 0.02

**Table 6:** Critical material properties for each zone of the FFF process and comments on each property based on experimental observation and from prior studies.

Zone	Material Property	Comments
Feed	Filament stiffness	A very stiff filament will not permit winding onto spools. Therefore, the filament remains in the vertical axis and length will be limited by room height or other obstructions. Above a certain stiffness, feed length will be determined by height, which can self-support weight. For pre-screening, material stiffness can be measured in a number of different modes, tensile, flexural, or torsion. We utilized a DMA in the single cantilever, but a universal tester (tensile, flexural, and torsion) or a texture analyser can also be used [13,73]. Zhang et al. [13] allocated the breaking stress as a quantification of filament stiffness as tested by using a texture analyzer.
	Filament brittleness	Brittle filaments can snap in the driving gears and prevent feeding. Brittleness (B) can be calculated from strain-at-break (ε_b_) and storage modulus (E′) using the Brostow-Hagg Lobland-Narkis Equation (see Equation (2)) for brittleness [37]. Primarily elongation-at break (%) is the value calculated for ε_b_ and the values are obtained from tensile testing if the correct test specimens are available [74]. Our modified approach was to test filament lengths to obtain strain-at-break from 3-point bending directly. Others have performed similar tests but solely defined the strain-at-break data as a brittleness measurement [13,73]. We calculate that B should be less than 0.0002 %Pa for materials to make suitable filaments.
	Column strength	Since most filaments act as a piston on the melt-front in the liquefier, the ability of the filament to withstand compressive force without buckling is an important variable [68,75]. Venkataraman et al. [68] determined a critical ratio for ceramic-based filaments above which a filament will withstand buckling. The ratio states that, if the elastic modulus of the filament is greater than the apparent viscosity by 3–5 × 10^5^ s^−1^ then the filament will maintain sufficient column strength during printing. Most thermoplastic materials will maintain the critical ratio [75], but it is a useful pre-screening tool for untypical materials or highly-filled materials.
	Filament softness	Soft materials can be squeezed between driving gears, which would limite or prevent feeding. Material hardness can be measured a number of ways, but the Shore durometer method is the most common approach [76].
	Dimensional consistency	Filament consistency will determine the feed rate to the heating end. Consistency is more than just a measure of filament diameter and can include ovality, pockmarks, gaps, and general deformities. Visual inspection is sufficient for eliminating the majority of the irregular filament.
	Filament diameter	Diameter ultimately determines feed rate to the heating end. Inconsistent filament diameter will result in inconsistent deposition and poor prints. Extrusion flow surging is a problem that occurs due to fluctuations in the feed or transition zone in the extrusion process [45]. A melt pump will eradicate the problem and produce a uniform filament but at added capital cost. Consistent material feeding and a correct temperature profile that permits stable melt formation can eliminate most surging. Die design can reduce the phenomenon and a longer land length by promoting a consistent melt output. Filament diameter is best measured at the point of filament production using laser micrometers or ultrasonic gauges.
Hot	Melt viscosity	As material softens and begins to melt, feeding from the melt to the nozzle is dependent on the back pressure formed due to the action of the driving gears forcing the filament downwards. High viscosity and the back pressure will be insufficient to force the melt through the nozzle die. Too high a force can lead to buckling of the filament [68]. Low viscosity and too much material will be pushed through the nozzle by preventing proper deposition. Melt viscosity is determined by a rheometer. A capillary rheometer at low shear is best suited since it most closely resembles the FFF extruder setup.
	Softening	Filament entering past the driving gear acts as a piston on the molten polymer below and, therefore, must maintain sufficient stiffness before melting to create the required back pressure. If the filament softens too soon, piston action efficiency will decrease and hinder melt deposition. A DMA storage modulus curve is a good representation of the stiffness of the material over an elevated temperature range.
Deposition	Melt flow rate	Melt flow rate is related to viscosity and is temperature dependent. High flow rate materials will more easily be pushed through the liquefier and nozzle. Too high and melt deposition will be uncontrollable. Low flow rate materials will be harder to push through the liquefier and nozzle. Too low of a flow rate and melt deposition becomes unachievable. The melt flow rate is determined by a melt flow indexer. Wang et al. [77] have recently determined that commercial filament grades should be greater than 10 g/10 min to achieve acceptable print quality.
	Melt feed consistency	The homogeneous flow of material is a critical necessity for a successful 3DP part. Surge feeding or starvation of material result in imperfection in the part’s building process. Most common signs of feed inconsistency are missing layers, layers misalignment, weak infill, low dimensional accuracy, and layer splitting. Feedstock material with consistent dimensions is crucial.
	Coalescence	Poor layer coalescence leads to inconsistencies in the structure of the printed parts, which creates critical points of failure, poor performance, and geometrical discrepancies. Coalescence increases with decreases in melt viscosity since there is greater polymer chain mobility and intermingling between layers [78]. Therefore, poor interlayer adhesion may be improved through higher printing temperatures. If deposited layers fail to adhere, print quality suffers considerably. Finished parts with the strong layer-to-layer union will possess higher mechanical toughness [79].
	Shrinkage and Warpage	Parts with subpar adhesion to the printing bed could exhibit warping due to deposited layers cooling down and contracting because of internal stresses, which results in partial deformation. If material fails to stick properly to the printing bed, a higher printing bed temperature might be necessary. The use of Kapton tape or Scotch™ blue painters tape improves the adhesion of materials to the printing bed and protects the bed from scratches. Environmental conditions, such as room temperature, should be taken into consideration when dealing with poor adhesion or warping since thermal gradients are the primary cause of internal stress [70]. Correction factors can be applied at the design stage to accommodate for known print shrinkage of specific materials. These factors are prevalent for common materials and are a common feature of 3D printing software. Kaveh 2015 et al. [80] describe a means for determining correction factors for material through the printing of a series of cubes, cylinders, and stairs.
	Moisture content	Trapped water will evaporate by exiting the nozzle and creating bubbles inside the extruded material, which disrupts the steady deposition of layers [81]. When using hygroscopic materials for long printing processes, it is important to consider the storage conditions of the feedstock material used for manufacturing. Production could fail due to absorption of moisture by the material. Adequate drying procedures should be adopted for an improperly stored filament.
	Die swell	Die swell is a well-established issue in polymer extrusion. The phenomena relates to the exiting diameter of the extrudate being greater than the diameter of the die and is related to the viscoelastic nature of the polymer. Die swell increases with increasing polymer molecular weight. It will affect the quality of the final print since it reduces the dimensional accuracy of the deposited layer. Die swell from the liquefier nozzle may be reduced through changes to the material formulation or changes in the nozzle design. However, the short land length of FFF printer nozzles may preclude the latter option. The primary means of dealing with die swell is to accommodate the design by specifying the deposited layer thickness to be 1.2–1.5 times the nozzle die diameter [82]. Material die swell can be measured using a capillary die rheometer [83].
